# NfL reliability across laboratories, stage-dependent diagnostic performance and matrix comparability in genetic FTD: a large GENFI study

**DOI:** 10.1136/jnnp-2023-332464

**Published:** 2024-01-19

**Authors:** Christoph Linnemann, Carlo Wilke, David Mengel, Henrik Zetterberg, Carolin Heller, Jens Kuhle, Arabella Bouzigues, Lucy L Russell, Phoebe H Foster, Eve Ferry-Bolder, John Cornelis Van Swieten, Lize C Jiskoot, Harro Seelaar, Fermin Moreno, Barbara Borroni, Raquel Sánchez-Valle, Daniela Galimberti, Robert Laforce, Caroline Graff, Mario Masellis, Maria Carmela Tartaglia, James Benedict Rowe, Elizabeth Finger, Rik Vandenberghe, Alexandre de Mendonca, Chris R Butler, Alexander Gerhard, Simon Ducharme, Isabelle L E Ber, Pietro Tiraboschi, Isabel Santana, Florence Pasquier, Johannes Levin, Markus Otto, Sandro Sorbi, Jonathan Daniel Rohrer, Matthis Synofzik

**Affiliations:** 1 Division Translational Genomics of Neurodegenerative Diseases, Hertie-Institute for Clinical Brain Research and Center of Neurology, University of Tübingen, Tübingen, Germany; 2 Center of Old Age Psychiatry, Psychiatric University Hospital (UPK), University of Basel, Basel, Switzerland; 3 Center for Neurodegenerative Diseases (DZNE), Tübingen, Germany; 4 UK Dementia Research Institute at UCL, London, UK; 5 Department of Psychiatry and Neurochemistry, Institute of Neuroscience and Physiology, The Sahlgrenska Academy at the University of Gothenburg, Mölndal, Sweden; 6 Dementia Research Centre, Department of Neurodegenerative Disease, UCL Queen Square Institute of Neurology, London, UK; 7 Neurologic Clinic and Policlinic, MS Center and Research Center for Clinical Neuroimmunology and Neuroscience Basel (RC2NB), University Hospital Basel, University of Basel, Basel, Switzerland; 8 Department of Neurology, Erasmus Medical Centre, Rotterdam, Netherlands; 9 Cognitive Disorders Unit, Department of Neurology, Donostia Universitary Hospital, San Sebastian, Spain; 10 Biodonostia Health Research Institute, Neuroscience Area, San Sebastian, Spain; 11 Neurology Unit, Department of Clinical and Experimental Sciences, University of Brescia, Brescia, Italy; 12 Alzheimer’s Disease and Other Cognitive Disorders Unit, Neurology Service, Hospital Clínic, Institut d’Investigacións Biomèdiques August Pi I Sunyer, University of Barcelona, Barcelona, Spain; 13 IRCCS Ospedale Policlinico, Fondazione Ca’ Granda, Milan, Italy; 14 Centro Dino Ferrari, University of Milan, Milan, Italy; 15 Clinique Interdisciplinaire de Mémoire, Département des Sciences Neurologiques, CHU de Québec, and Faculté de Médecine, Université Laval, Québec, Alberta, Canada; 16 Center for Alzheimer Research, Division of Neurogeriatrics, Department of Neurobiology, Care Sciences and Society, Bioclinicum, Karolinska Institutet, Solna, Sweden; 17 Unit for Hereditary Dementias, Theme Aging, Karolinska University Hospital, Solna, Sweden; 18 Sunnybrook Health Sciences Centre, Sunnybrook Research Institute, University of Toronto, Toronto, Ontario, Canada; 19 Tanz Centre for Research in Neurodegenerative Diseases, University of Toronto, Toronto, Ontario, Canada; 20 Department of Clinical Neurosciences, University of Cambridge, Cambridge, UK; 21 Department of Clinical Neurological Sciences, University of Western Ontario, London, Ontario, Canada; 22 Laboratory for Cognitive Neurology, Department of Neurosciences, KU Leuven, Leuven, Belgium; 23 Neurology Service, University Hospitals Leuven, Leuven, Belgium; 24 Faculty of Medicine, University of Lisbon, Lisbon, Portugal; 25 Nuffield Department of Clinical Neurosciences, Medical Sciences Division, University of Oxford, Oxford, UK; 26 Department of Brain Sciences, Imperial College, London, UK; 27 Division of Neuroscience and Experimental Psychology, Wolfson Molecular Imaging Centre, University of Manchester, Manchester, UK; 28 Department of Geriatric Medicine, Klinikum Hochsauerland, Arnsberg, Germany; 29 Department of Psychiatry, McGill University Health Centre, McGill University, Montreal, Québec, Canada; 30 McConnell Brain Imaging Centre, Montreal Neurological Institute, McGill University, Montreal, Québec, Canada; 31 Sorbonne Université, Paris Brain Institute – Institut du Cerveau – ICM, Inserm U1127, CNRS UMR 7225, AP-HP, Hôpital Pitié-Salpêtrière, Paris, France; 32 Centre de référence des démences rares ou précoces, IM2A, Département de Neurologie, AP-HP, Hôpital Pitié-Salpêtrière, Paris, France; 33 Istituto Neurologico Carlo Besta, Fondazione IRCCS, Milan, Italy; 34 Neurology Service, Faculty of Medicine, University Hospital of Coimbra (HUC), University of Coimbra, Coimbra, Portugal; 35 Center for Neuroscience and Cell Biology, Faculty of Medicine, University of Coimbra, Coimbra, Portugal; 36 University of Lille, Lille, France; 37 Inserm 1172, Lille, France; 38 Department of Neurology, Ludwig-Maximilians Universität München, Munich, Germany; 39 German Center for Neurodegenerative Diseases (DZNE), Munich, Germany; 40 Department of Neurology, University of Ulm, Ulm, Germany; 41 Department of Neurofarba, University of Florence, Firenze, Italy; 42 Don Carlo Gnocchi, IRCCS Fondazione, Firenze, Italy

**Keywords:** COGNITION, FRONTOTEMPORAL DEMENTIA, NEUROPSYCHIATRY

## Abstract

**Background:**

Blood neurofilament light chain (NfL) is increasingly considered as a key trial biomarker in genetic frontotemporal dementia (gFTD). We aimed to facilitate the use of NfL in gFTD multicentre trials by testing its (1) reliability across labs; (2) reliability to stratify gFTD disease stages; (3) comparability between blood matrices and (4) stability across recruiting sites.

**Methods:**

Comparative analysis of blood NfL levels in a large gFTD cohort (GENFI) for (1)–(4), with n=344 samples (n=148 presymptomatic, n=11 converter, n=46 symptomatic subjects, with mutations in *C9orf72*, *GRN* or *MAPT*; and n=139 within-family controls), each measured in three different international labs by Simoa HD-1 analyzer.

**Results:**

NfL revealed an excellent consistency (intraclass correlation coefficient (ICC) 0.964) and high reliability across the three labs (maximal bias (pg/mL) in Bland-Altman analysis: 1.12±1.20). High concordance of NfL across laboratories was moreover reflected by high areas under the curve for discriminating conversion stage against the (non-converting) presymptomatic stage across all three labs. Serum and plasma NfL were largely comparable (ICC 0.967). The robustness of NfL across 13 recruiting sites was demonstrated by a linear mixed effect model.

**Conclusions:**

Our results underline the suitability of blood NfL in gFTD multicentre trials, including cross-lab reliable stratification of the highly trial-relevant conversion stage, matrix comparability and cross-site robustness.

## Introduction

Genetic frontotemporal dementias (gFTDs) represent a group of progressive neurodegenerative diseases characterised by a progressive decline of executive, behavioural and language functions, frequently resulting from mutations in the genes chromosome open reading frame 72 (*C9orf72*), progranulin (*GRN*) or microtubule-associated protein tau (*MAPT*).^1^ Neurofilament light chain (NfL)—an intermediate filament that constitutes part of the neuronal cytoskeleton—is released after neuronal damage into the interstitial fluid, cerebrospinal fluid and blood. Blood-based NfL has an increasing impact as a trial biomarker in gFTD for multiple contexts of use, for example, patient stratification,^2–5^ trial inclusion,^6^ toxicity monitoring and treatment-response capture,^7^ and has now been approved by the U.S. Food and Drug Administration as a surrogate endpoint contributing to approval of novel drugs (tofersen).^8^ However, its wider use in multicentre trials—as well as in real-world clinical settings—has been questioned due to potential cross-laboratory heterogeneity in analytical approaches and blood sample matrices that might lead to different, non-comparable concentrations of blood NfL.^9 10^

Leveraging a large gFTD cohort, we here aimed to facilitate the use of blood NfL in gFTD multicentre trials and real-world clinical settings by testing: (1) its reliability across laboratories, measured at different time points, by different end-user devices and kits; (2) cut-off values maximising stratification accuracy of the trial relevant gFTD disease stages (conversion stage, symptomatic stage), with cut-off values validated across labs; (3) comparability between blood matrices and (4) robustness across recruiting sites.

## Methods

### Cohort and NFL measurements

Subjects were patients with FTD caused by mutations in the genes *C9orf72*, *GRN* or *MAPT* (symptomatic mutation carriers), and their respective first-degree relatives (ie, either presymptomatic mutation carriers or noncarriers serving as within-family controls), recruited by the international Genetic FTD Initiative (GENFI; www.genfi.org.uk)^11^ at 13 sites. The comparative analysis included n=344 blood samples (n=148 from presymptomatic carriers; n=11 from carriers that converted during the observation period; n=46 from symptomatic carriers; n=139 from within-family-controls; for characteristics of these subcohorts, see online supplemental table 1 that were independently measured for NfL levels by Single molecule array (Simoa; HD-1 analyzer, Quanterix, Billerica, Massachusetts, USA) in three different laboratories (lab 1: Basel, Switzerland^5^; lab 2: Rotterdam, the Netherlands^4^; lab 3: London, UK^2^), using different NfL kits (Basel and Rotterdam: NF-Light Advantage Kit 103186 (V.1); London: Neurology 4-Plex A Kit 102153), according to the manufacturer’s instructions. The blood matrices for NfL analysis were serum (Basel and Rotterdam) and plasma (London). Further methodological details of NfL measurements, details of the GENFI protocol, participant demographics, clinical classification of the disease stages (ie, presymptomatic carriers, converters, symptomatic carriers) as well as NfL quantification were described elsewhere.^2 4 5 11^

10.1136/jnnp-2023-332464.supp1 Supplementary data

### Statistical analyses

SPSS for Windows V.29.0 (IBM), Sigmaplot for Windows V.15 (Inpixion, Germany) and RStudio 2022.07.2 were used for statistical analyses. NfL values were not normally distributed and therefore log-transformed. For age-corrected z-scores—taking into consideration the age-related NfL increase observed in controls—log-transformed NfL values were normalised relative to their distribution in controls.^5^ The consistency of NfL measurements across the three different labs was quantified by intraclass correlation coefficients (ICC; two-way mixed effect model, single measures, absolute agreement^12^). Bland-Altman analyses^13^ were used to quantify between-lab bias, defined as the mean of the differences; limits of agreement, that is, the mean of the bias±1.96 times the SD of the differences; and 95% CIs for the bias with lower and upper limits of agreement. The diagnostic performance of NfL was assessed by receiver operating characteristic (ROC) analysis^14^ and calculating areas under the curve (AUCs), as well as optimal operating points, that is, cut-off values (assuming a cost ratio of 1 and a pretest probability of 0.5), maximising stratification accuracy for different gFTD disease stages. The predictive value for an NfL-based disease stage stratification was addressed by calculating positive and negative likelihood ratios (LR+ and LR−).^15^ Linear mixed effect models were used to characterise the stability of log-transformed NfL levels across recruiting sites (with categorial factors of disease stage and genetic status, and metric covariate of age as fixed effects).

## Results

**Figure 1:**
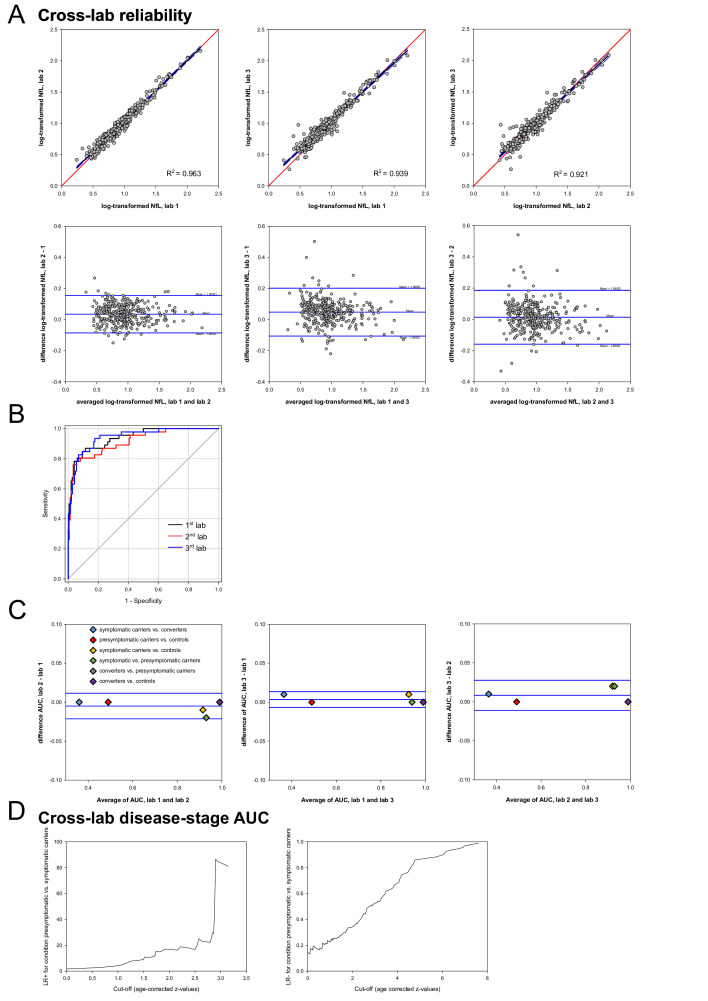
Cross-lab reliability, cross-lab disease-stage cut-offs and likelihood ratios (LR) and blood matrix comparability in genetic FTD. (A) Reliability of blood NfL measurements in genetic FTD (gFTD) across three labs (lab 1 and 2 serum, lab 3 plasma)—linear regressions and Bland-Altman analyses of log-transformed NfL values. For detailed statistics, see online supplemental table 2. (B) Comparative across-lab analysis of ROC curves and AUC values for the condition ‘presymptomatic versus symptomatic carriers’. Detailed values of AUC±SE and 95% CI are given in the Results section. (C) Reliability of AUC values across three labs—Bland-Altman analyses for all stage comparisons. For detailed statistics, see online supplemental table 2. (D) Prediction of individual risk factors at different cut-offs for the condition ‘presymptomatic versus symptomatic carriers’ (age-corrected z-values, first lab) by positive (LR+) and negative (LR−). AUC, area under the curve; FTD, frontotemporal dementia; NfL, neurofilament light chain; ROC receiver operating characteristic

NfL levels showed an excellent consistency across the three labs (ICC 0.964, 95% CI lower to upper limit 0.946 to 0.974), as demonstrated by a two-way mixed effect model. Reliability of NfL levels was high and bias was low across all three labs, as shown by linear regressions and Bland-Altman analyses with a maximal bias±SD of 1.12 pg/mL±1.20 (for summary, see figure 1A). The performance of blood NfL to serve as a disease stage stratification biomarker in gFTD was investigated by ROC curve analyses and calculation of optimal cut-off values maximising stratification accuracy for different gFTD disease stages. Blood NfL allowed discrimination of conditions (1) symptomatic carriers versus controls (AUC: 0.91; cut-off, given as z-value: 2.83), (2) converters versus controls (AUC: 0.89; cut-off z: 3.05), (3) converters versus presymptomatic carriers (AUC: 0.86; cut-off z: 3.19) and (4) symptomatic versus presymptomatic carriers (AUC: 0.88; cut-off z 3.20). NfL did not allow a discrimination of (5) presymptomatic carriers versus controls (AUC: 0.57) or (6) symptomatic carriers versus converters (AUC: 0.59), with AUCs close to the random classifier level (for detailed results, see table 1). Concordance of AUCs across the three labs for all comparisons was high (maximum difference±SE 0.02±0.01), as exemplified in figure 1B for the discrimination of symptomatic vs presymptomatic carriers (lab 1 AUC±SE 0.94±0.02, 95% CI 0.90 to 0.98; lab 2 AUC±SE 0.92±0.02, 95% CI 0.86 to 0.96; lab 3 AUC±SE 0.94±0.02, 95% CI 0.91 to 0.98)). The high reliability of AUC across labs for all disease stage comparisons was further corroborated by Bland-Altman analysis (see figure 1C), with a maximal bias of 0.01±0.01 (AUC±SD). For a genotype-specific analysis (C9orf72, MAPT, GRN) of NfL cross-lab reliability and disease-stage AUC, see online supplemental figures 1,2 and online supplemental tables 3,4.

**Table 1:** Receiver operating characteristic (ROC) curve analysis with areas under the curve (AUC) and optimal cut-offs for separating different gFTD stages and conditions

gFTD conditions	AUC	SE	Optimal cut-off		
			z-value	Specificity (%)	Sensitivity (%)
Symptomatic carriers versus controls	0.91	0.02	2.83	100	49.5
Converters versus controls	0.89	0.07	3.05	100	42.9
Converters versus presymptomatic carriers	0.86	0.08	3.19	100	42.9
Symptomatic versus presymptomatic carriers	0.88	0.02	3.20	100	46.2
Presymptomatic carriers versus controls	0.57	0.03			
Symptomatic carriers versus converters	0.59	0.10			

Data from lab 1; optimal cut-off values are given as z-values (corrected for age). For the data from lab 2 and 3, see figure 1B and C.

gFTD, genetic frontotemporal dementia.

The disease stage-specific stratification value of NfL levels—beyond dichotomising cut-offs—was demonstrated by LR (see figure 1D). For exemplary illustration of the individual risk prediction of being presymptomatic versus symptomatic carrier at different NfL levels by LR+ and LR− see figure 1C (NfL values from lab 1). An NfL z-value of 3 corresponded to an LR+ of 83 and an LR− of 0.5.

NfL values in serum and plasma (n=344 samples of each matrix) were largely comparable (ICC 0.967, 95% CI lower to upper 0.894 to 0.977), as calculated by a two-way mixed effect model. The median ratio serum/plasma was 0.95.

The high robustness of NfL across 13 recruiting sites was shown by a linear mixed effect model, as the categorial variable ‘recruiting site’ did not explain any variance (estimate 0.001, SE 0.001, Wald-Z 1.403, significance 0.161).

## Discussion

Blood NfL has an increasing impact as a trial biomarker in gFTD for multiple contexts of use^5 7^ and is now being increasingly acknowledged by the FDA as a surrogate endpoints in drug approval processes.^8^ However, its wider use in multicentre trials and real-world clinical settings is limited by lack of larger data demonstrating cross-lab reliability, cross-lab validated cut-off values and cross-lab validated comparability between blood matrices in gFTD. Leveraging a large genetic FTD, our findings show that blood NfL is a biomarker in gFTD with high reliability across labs—even if assessed at different time points, and by partly different kits (NF-Light Advantage Kit vs Neurology 4-Plex A Kit). This finding confirms and extends earlier findings showing a good cross-lab reliability of blood NfL, which so far, however, has been limited to smaller sample sets and non-gFTD cohorts.^16^ Given, however, that all three labs in our study still used the same type of platform (Simoa HD-1), future studies need to investigate a potential decrease in cross-lab reliability if different measurement platforms are being used for blood NfL (eg, Ella,^17^ Uman,^18^ Atellica^19^). A pilot study on this showed promising results.^20^

Reliable cut-off values of blood NfL for accurately stratifying different gFTD disease stages are key for its use as a molecular stratification marker of gFTD subjects into treatment trials.^3 5 7^ In particular, reliable blood-based stratification of subjects close to conversion to the symptomatic phase of the disease will be of extremely high value to identify and recruit subjects into upcoming mechanistic treatment trials tailored to prevent neurodegeneration by early intervention.^5 21^ Extending earlier findings on blood NfL cut-offs in gFTD,^3^ our findings now indicate that these cut-off values can be provided by blood NfL for gFTD even with a high reliability across labs. In addition, they also show that NfL levels in converting carriers are already more similar to symptomatic carriers than (non-converting) presymptomatic carriers. Nevertheless, in the absence of a certified reference material, value assigned by a certified reference method, the reported cut-offs remain preliminary and prospective laboratory-specific validation remains required.

Multicentre use of blood NfL—whether in trials or real-world clinical settings—is inherently characterised by cross-centre variability in preanalytical sample handling. Our data from a large set of different sites (n=13) suggest that this variability might not exert a substantial effect on multicentre blood NfL values—even despite the fact that no strictly enforced cross-centre harmonised standard operating procedure or centralised biosampling monitoring had been employed across centres. These data corroborate blood NfL as a very stable biomarker that is resistant to most types of clinically relevant variation in preanalytical sample handling.^22^ Future studies with larger sample batches per centre and testing more extreme variabilities in preanalytical sample handling are warranted to further investigate and specify the limits of this cross-centre comparability.

Real-world clinical multicentre use of blood NfL moreover often faces the challenge that samples come from different blood matrices (eg, serum vs plasma).^9^ While our findings confirm differences in the absolute blood NfL concentrations between serum and plasma, they at the same time show a high consistency between both blood matrices, allowing comparability of both matrices. The calculated median ratio serum/plasma might be a first coarse help when comparing results derived from these different matrices. However, its use might be limited to Simoa-based blood NfL measurements, and further larger in-depth studies in independent cohorts are required to confirm this factor.

Our study has several limitations. First, although leveraging the largest gFTD cohort existing so far, the sample size is partly limited by the requirement to measure each sample in three labs, leading to limited sample sizes in particular for some gFTD subcohorts (eg, converters). Second, the construct and wording of ‘cut-offs’ suggest a separating dichotomy where in fact a biological continuum of NfL levels and disease progression exists.

Despite these limitations, our results underline the suitability of blood NfL as a fit-for-purpose biomarker in gFTD multicentre trials.
