# Supplementary material for: NfL reliability across laboratories, stage-dependent diagnostic performance and matrix comparability in genetic FTD: a large GENFI study
Source: J Neurol Neurosurg Psychiatry. 2024 Jan 19;95(9):822–8. doi: 10.1136/jnnp-2023-332464 (PMC11347244; doi:10.1136/jnnp-2023-332464)
Supplement: Supplementary data [file jnnp-2023-332464supp001.pdf]

Supplementary Figures

Cross-lab reliability for each genotype

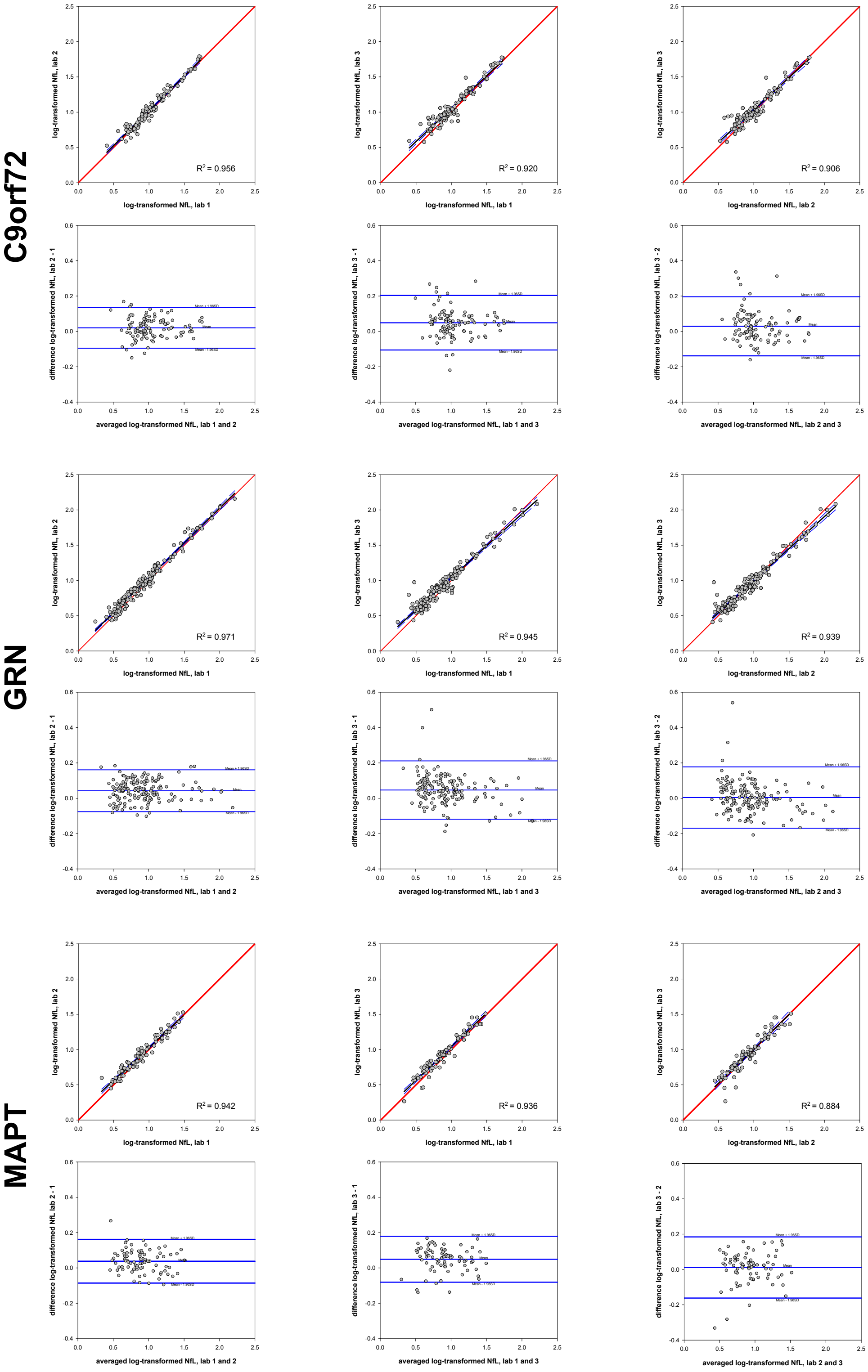

Supplementary Figure 1: Cross-lab reliability for each genotype (*C9orf72*, *GRN*, and *MAPT*) of blood NfL measurements in genetic FTD (gFTD) across three labs (lab 1 and 2 serum, lab 3 plasma) - linear regressions and Bland-Altman analyses of log-transformed NfL values. For detailed statistics see Supplementary Table 3.

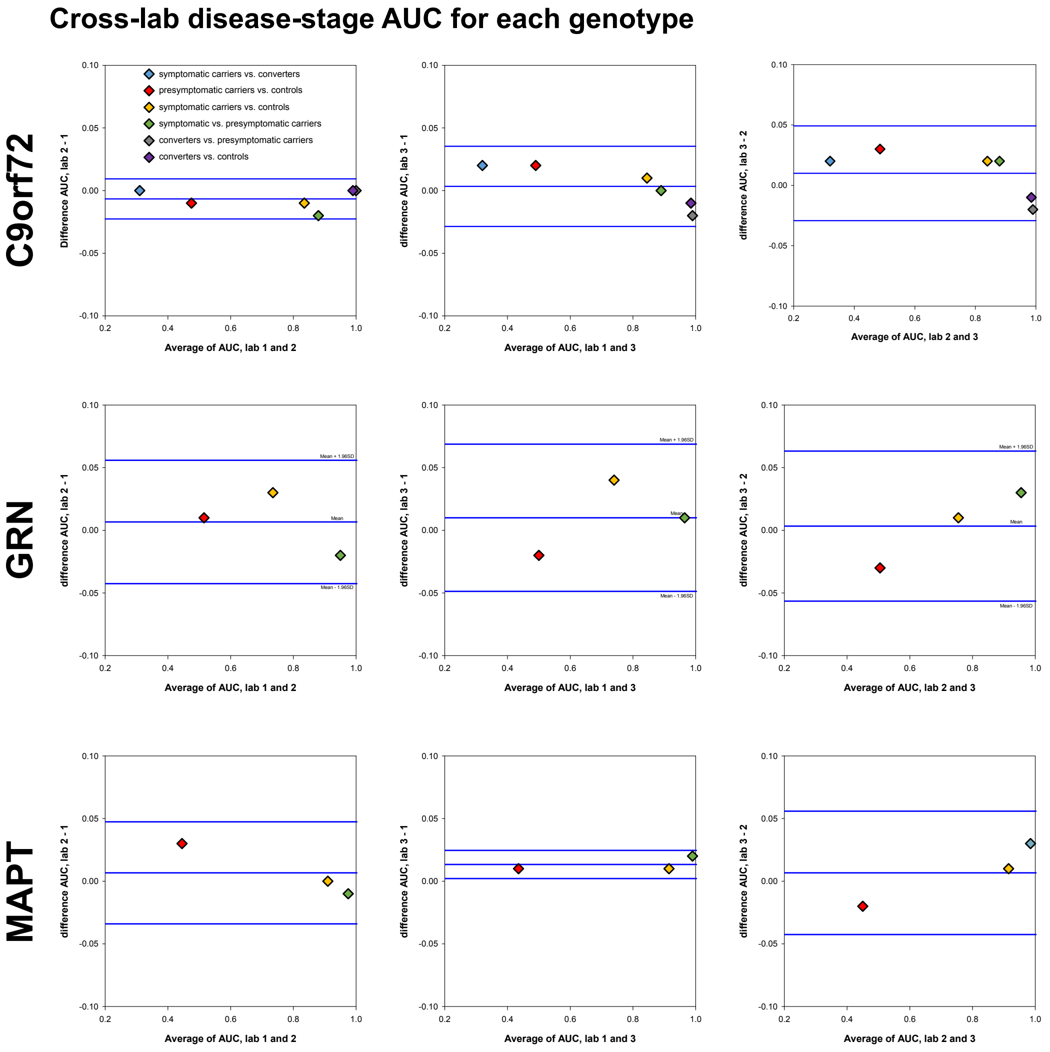

Supplementary Figure 2: Cross-lab disease stage AUC for each genotype (*C9orf72*, *GRN*, and *MAPT*) of blood NfL measurements in genetic FTD (gFTD) across three labs - Bland-Altman analyses of disease stage AUC. For detailed statistics see Supplementary Table 4. An analysis for the converters was performed for C9orf72, but not for GRN and MAPT due to the sample size (see cohort characteristics in Supplementary Table 1).

Supplementary Tables

Group	Subjects	Subjects with longitudinal samples	Male sex	Age (years)	Disease onset (years)
Presymptomatic	66	57	42	41 (34.00 – 49.38)	n.a.
– C9orf72	22	17	14	42 (35.04 – 48.88)	n.a.
– GRN	29	28	16	43 (34.97 – 52.62)	n.a.
– MAPT	15	12	12	36 (32.17 – 46.44)	n.a.
Converter	4	4	1	65 (59.72 – 67.53)	n.a.
– C9orf72	3	3	1	63 (56.89 – 65.00)	n.a.
– GRN	1	1	0	68	n.a.
Symptomatic	21	18	8	63 (59.29 – 66.23)	56 (53.00 – 60.00)
– C9orf72	7	6	1	66 (64.17 – 71.66)	57 (51.50 – 61.50)
– GRN	8	7	4	61 (59.67 – 66.03)	58 (54.75 – 58.50)
– MAPT	6	5	3	61 (58.30 – 64.51)	54 (52.25 – 55.50)
Controls (non-carriers)	60	53	34	45 (37.52 – 55.74)	

Supplementary Table 1. Cohort characteristics at baseline. Data are reported as median and interquartile range. N.a.: not applicable.

A,				
		Lab 1 vs. lab 2	Lab 1 vs. lab 3	Lab 2 vs. lab 3
	Bias	1.08	1.12	1.03
	Std Dev	1.15	1.20	1.22
	Limits of Agreement	0.82, 1.43	0.78, 1.59	0.69, 1.53
	Bias CI 95% CI	1.07 to 1.10	1.10 to 1.14	1.01 to 1.05
	Lower Limit of Agreement CI 95% CI	0.80 to 0.84	0.76 to 0.81	0.67 to 0.72
	Upper Limit of Agreement CI 95% CI	1.39 to 1.47	1.54 to 1.64	1.48 to 1.59
B.				
	Bias	-0.1	0.00	0.01
	Std Dev	0.01	0.01	0.01
	Limits of Agreement	-0.21, to 0.01	-0.07, to 0.01	-0.01, to 0.03
	Bias CI 95% CI	-0.01 to 0.00	-0.00 to 0.01	-0.00 to 0.02
	Lower Limit of Agreement CI 95% CI	-0.04 to -0.01	-0.02 to 0.00	-0.03 to 0.01
	Upper Limit of Agreement CI 95% CI	-0.01 to 0.03	0.00 to 0.02	0.01 to 0.05

Supplementary Table 2. Numerical results of the Bland-Altman analysis comparing blood NfL measurements in genetic FTD across three labs (lab 1 and 2 serum, lab 3 plasma) (A: see Figure 1A; B: see Figure 1C). Data are given as pg/ml.

C9orf72				
		Lab 1 vs. lab 2	Lab 1 vs. lab 3	Lab 2 vs. lab 3
	Bias	1.58	1.12	1.07
	Std Dev	1.15	1.20	1.23
	Limits of Agreement	0.11, 1.38	0.78, 1.58	0.72, 1.58
	Bias CI 95% CI	1.02 to 1.07	1.07 to 1.15	1.02 to 1.12
	Lower Limit of Agreement CI 95% CI	0.76 to 0.83	0.74 to 0.83	0.68 to 0.78
	Upper Limit of Agreement CI 95% CI	1.32 to 1.45	1.51 to 1.70	1.48 to 1.70
GRN				
	Bias	1.10	1.12	1.00
	Std Dev	1.15	1.20	1.23
	Limits of Agreement	0.83, 1.45	0.76, 1.62	0.68, 1.51
	Bias CI 95% CI	1.07 to 1.12	1.07, to 1.14	0.98 to 1.05
	Lower Limit of Agreement CI 95% CI	0.81 to 0.87	0.72 to 0.79	0.63 to 0.71
	Upper Limit of Agreement CI 95% CI	1.38 to 1.51	1.55 to 1.70	1.41 to 1.58
MAPT				
	Bias	1.10	1.12	1.02
	Std Dev	1.15	1.17	1.23
	Limits of Agreement	0.81, 1.45	0.83, 1.51	0.69, 1.55
	Bias CI 95% CI	1.05 to 1.12	1.07 to 1.15	0.98 to 1.07
	Lower Limit of Agreement CI 95% CI	0.78 to 0.87	0.78 to 0.87	0.63 to 0.74
	Upper Limit of Agreement CI 95% CI	1.38 to 1.55	1.41 to 1.62	1.41 to 1.66

Supplementary Table 3. Numerical values of Bland-Altman analysis for cross-lab reliability for each genotype (see supplementary figure 1). Data are given as pg/ml.

C9orf72				
		Lab 1 vs. lab 2	Lab 1 vs. lab 3	Lab 2 vs. lab 3
	Bias	-0.07	0.03	0.01
	Std Dev	0.01	0.02	0.02
	Limits of Agreement	-0.02, 0.01	-0.03, 0.04	-0.03, 0.05
	Bias CI 95% CI	-0.02 to 0.00	-0.02 to 0.02	-0.01 to 0.03
	Lower Limit of Agreement CI 95% CI	-0.04 to -0.01	-0.06 to 0.00	-0.07 to 0.01
	Upper Limit of Agreement CI 95% CI	-0.01 to 0.03	0.00 to 0.07	0.01 to 0.09
GRN				
	Bias	0.01	0.01	0.00
	Std Dev	0.03	0.03	0.03
	Limits of Agreement	-0.04, 0.06	-0.05, 0.07	-0.06, 0.06
	Bias CI 95% CI	-0.18 to 0.20	-0.21, to 0.23	-0.22 to 0.23
	Lower Limit of Agreement CI 95% CI	-0.36 to 0.28	-0.43 to 0.33	-0.44 to 0.33
	Upper Limit of Agreement CI 95% CI	-0.26 to 0.38	-0.31 to 0.45	-0.33 to 0.45
MAPT				
	Bias	0.01	0.01	0.01
	Std Dev	0.02	0.06	0.03
	Limits of Agreement	-0.03, 0.05	0.00, 0.02	-0.04, 0.06
	Bias CI 95% CI	-0.15 to 0.16	-0.03 to 0.06	-0.18 to 0.20
	Lower Limit of Agreement CI 95% CI	-0.30 to 0.23	-0.07 to 0.08	-0.36 to 0.28
	Upper Limit of Agreement CI 95% CI	-0.22 to 0.31	-0.05 to 0.10	-0.26 to 0.38

Supplementary Table 4. Numerical values of Bland-Altman analysis for cross-lab disease-stage AUC for each genotype (see supplementary figure 2). Data are given as pg/ml.
